# Modification Targeting the “Rana Box” Motif of a Novel Nigrocin Peptide From *Hylarana latouchii* Enhances and Broadens Its Potency Against Multiple Bacteria

**DOI:** 10.3389/fmicb.2018.02846

**Published:** 2018-11-28

**Authors:** Kaifan Bao, Weiyuan Yuan, Chengbang Ma, Xi Yu, Lei Wang, Min Hong, Xinping Xi, Mei Zhou, Tianbao Chen

**Affiliations:** ^1^Jiangsu Key Laboratory for Pharmacology and Safety Evaluation of Chinese Materia Medica, Nanjing University of Chinese Medicine, Nanjing, China; ^2^Natural Drug Discovery Group, School of Pharmacy, Faculty of Medicine, Health and Life Sciences, Queen’s University Belfast, Belfast, United Kingdom; ^3^Nanjing Pharmaceutical Co., Ltd., Nanjing, China

**Keywords:** nigrocin, Rana box, modification, MRSA, antibiotic resistance, pneumonia

## Abstract

Public health is confronting the threat caused by antibiotic resistance and this means new antibacterial strategies must be developed urgently. Antimicrobial peptides (AMPs) have been considered as promising therapeutic candidates against infection in the post-antibiotic era. In this paper, we dismissed the significance of “Rana box” in the natural nigrocin-HL identified from skin secretion of *Hylarana latouchii* by comparing its activity with nigrocin-HLD without the motif. By substituting the “Rana box” sequence with an amidated phenylalanine residue, the natural peptide was modified into a shorter AMP nigrocin-HLM. Activities and toxicities of these two peptides *in vitro* and *in vivo* were compared. As a result, nigrocin-HLM not only displayed significantly increased potency against several representative microbes, but also high activity against the antibiotic-resistant methicillin-resistant *S. aureus* (MRSA, NCTC 12493 and ATCC43300 and several clinical isolates) as evidenced by markedly reduced minimal inhibitory concentration (MIC), minimal bactericidal concentration (MBC), and minimum biofilm eradication concentration (MBEC). More strikingly, nigrocin-HLM exhibited prominent inhibition against MRSA infection in a pneumonia mice model. In addition, the substitution attenuated the toxicity of nigrocin-HLM as evidenced by precipitously decreased hemolytic and cytotoxic activities *in vitro*, and acute toxicity to mice *in vivo*. Taken these results into consideration, nigrocin-HLM should be a promising therapeutic candidate for anti-infection. And in addition to dismiss an indispensable role of “Rana box” in maintaining antimicrobial activity of nigrocin-HL, our data provided an inspired strategy for peptide optimization.

## Introduction

Antibiotic resistance has disseminated rapidly because of multifactorial reasons including indiscriminate use and some complex ancient antibiotic-resistance mechanisms of bacteria (Brown and Wright, 2016; Dickey et al., 2017). To illustrate, as a developed strain of *Staphylococcus aureus* (*S. aureus*) through horizontal gene transfer and natural selection, MRSA could induce numerous infections of skin, bone, joint, soft-tissue, respiratory system and central nervous system (Liu et al., 2011; Gurusamy et al., 2013). To date, vancomycin has been considered as the best therapeutic agent to deal with infections caused by MRSA, but its disadvantages like extravascular diffusion and relatively slow action may lead to its decreased efficacy *in vivo* (Michel and Gutmann, 1997). Although the *in vitro* potency of combination treatment of vancomycin with some other antibiotics has been demonstrated, their mechanisms targeting specific phase of cell growth and proliferation may still permit MRSA to develop resistance to them. In consequence, it facilitates clinicians and researchers to develop new therapeutic approaches with broad-spectrum activity and selectivity to address the crisis.

Compared with conventional antibiotics, AMPs are reported to possess unique membrane-disrupting antimicrobial mode of actions represented by some structurally defined models including barrel-stave, toroidal and detergent-like carpet models, etc. (Mahlapuu et al., 2016). Furthermore, a majority of intracellular activity possessed by AMPs have been reported, which include inhibition of DNA, RNA and protein synthesis, protease, cell wall biosynthesis, cell metabolism and so on. Hence, it is logical that antimicrobial activity of an AMP could be a mixture of several modes of action happening concurrently, which makes pathogens virtually impossible to develop resistance to them in sharp contrast with traditional antibiotics (Fjell et al., 2011; Scocchi et al., 2016; Grassi et al., 2017; Le et al., 2017). Some motifs in AMPs have been considered as pivotal for them to possess antimicrobial activity and structural stability while some other studies refuted the indispensable roles of these highly conserved sequences. To illustrate, study on brevinin 1E amide suggested that the disulphide bridge was pivotal for the α-helical structure but not significant for its antimicrobial potency, as activity was unaffected even when cysteine residues are substituted by serine (Kwon et al., 1998). In contrast, the elimination of the heptapeptide motif in B1CTcu5, an AMP isolated from *Clinotarsus curtipes*, prominently reduced antibiotic and hemolytic activity of it (Abraham et al., 2015). Nigrocin is a representative anuran AMP family, and after sequence alignment between nigrocin-HL and other members of nigrocin family reported previously, a highly conserved heptapeptide motif “CGLXGLC” named “Rana box” at C-terminus was confirmed in the nigrocin family (Park et al., 2001; Conlon et al., 2006; Li et al., 2007; Che et al., 2008; Iwakoshi-Ukena et al., 2011b). However, it was not well determined if “Rana box” motif, as a widespread disulphide- bridged structure among anuran AMPs, was an indispensable domain for antimicrobial activity of these AMPs.

Here, we describe the isolation and identification of a novel AMP named nigrocin-HL from the skin secretion of *H. latouchii* using “shotgun” molecular cloning and mass spectrometry. By comparing the activity between the synthesized peptide without “Rana box” and the original peptide against several microbes *in vitro*, we dismissed the key role of this motif in maintaining the antimicrobial potency of nigrocin-HL. Moreover, an analog of it named nigrocin-HLM was designed and synthesized by “Rana box”-targeted and C-terminus amidation, which not only possessed activity against antibiotic-resistant bacteria, but showed much less toxicity both *in vitro* and *in vivo* as well.

## Materials and Methods

### Specimen Biodata and Secretion Acquisition

Specimens of the oriental broad-folded frog, *H. latouchii* (Synonym of *Sylvirana latouchii*), (sex undetermined; *n* = 12; 3.5–5.0 cm in snout-to-vent length) were captured in a mountainous region of Fujian Province, China. Skin secretions were induced by mild electrical transdermal stimulation of the dorsal skin, as described previously (Tyler et al., 1992). After stimulation, secretions were rinsed with deionized water, and maintained at 4°C prior to being snap frozen with liquid nitrogen, lyophilized and stored at -20°C for further analyses. All the procedures were subject to ethical approval and carried out under appropriate U.K. animal research personal and project licenses.

### “Shotgun” Cloning of Nigrocin-HL Precursor-Encoding cDNA

Molecular cloning of nigrocin-HL precursor-encoding cDNA from a skin secretion-derived cDNA library of *H. latouchii* were carried out as described by Gao et al. (2016) with minor changes as follow: To obtain the full-length peptide precursor nucleic acid sequence data, the 3’-RACE procedure was performed using a SMART-RACE kit (Clontech, Palo Alto, CA, United States) with a NUP primer (supplied with the kit) and a degenerate sense primer (S1; 5’-GAWYYAYYHRAGCCYAAADATGTTCA-3’) designed to a highly conserved domain of the 5′ untranslated region of previously characterized esculentin cDNAs from *Rana* species (Chen et al., 2006). Then, purified PCR products were cloned using a pGEM^®^-T easy vector system (Promega Corporation, United States) and sequenced by an ABI 3100 automated capillary sequencer (Applied Biosystems, Foster City, CA, United States).

### Peptide Modification

To probe the role of “Rana box” structure in nigrocins, the heptapeptide motif of nigrocin-HL was depleted to obtain a shorter peptide named nigrocin-HLD (Supplementary Figure S1). Taken activity improvement and toxicity reduction into consideration, another peptide nigrocin-HLM was designed by substituting the “Rana box” with a phenylalanine residue and C-terminus amidation. The analogs nigrocin-HLD and nigrocin-HLM and the original peptide were all synthesized by solid-phase Fmoc chemistry and purified by reverse phase HPLC (RP-HPLC) for further study.

### Determination of Secondary Structures of Peptides and Validation of Molecular Models

The I-TASSER and QUARK servers (Zhang, 2008; Xu and Zhang, 2012) were utilized to predict the secondary structures and deduce the 3D models of nigrocin-HL and its analog nigrocin-HLM. The 3D models with C-score >-1.5 were selected, which indicated that these models possessed correct global topology. Subsequently, the 3D models of the peptides were rendered with PyMOL (The EduPyMOL Molecular Graphics System, Version 2.0.2 Schrödinger, LLC). In addition, to correct the disulfide bridge of nigrocin-HL, the Swiss PDB Viewer (Guex and Peitsch, 1997) was used, as previously described by Porto et al. (2012) and Porto and Franco (2013). Briefly, the torsions of Gly19 were changed to approximate the Cys residues, then, the bonds between sulfur and β-carbon atoms were rotated to make the disulfide bridge. Subsequently, the GROMOS implementation of Swiss PDB Viewer was used for energy minimization, by 50,000 steps of steepest descent algorithm to remove eventual steric clashes.

Stereochemical quality of these 3D models were evaluated by PROCHECK (Morris et al., 1992). And PROSA was applied to validate model accuracy since it uses knowledge-based potentials of mean force and shows local model quality by plotting energies as a function of amino acid sequence position (Maganti et al., 2010).

### Prediction of Physiochemical Properties

A series of physiochemical properties of the peptides, including hydrophobicity, hydrophobic moments and net charge at neutral pH, and helical wheels of nigrocin-HL and nigrocin-HLM, were predicted by Heliquest^1^.

In addition, hydrophobicity of nigrocin-HL and nigrocin-HLM was further determined by RP-HPLC. Peptides (1 mg each) were dissolved in 0.5 ml trifluoroacetic acid (TFA)/water (0.5/99.95, v/v) (Buffer A) and 0.5 ml TFA/water/acetonitrile (0.5/19.95/80, v/v) (Buffer B). Supernatant was eluted from an analytical RP-HPLC Jupiter C5 column (250 nm × 4.6 mm, Phenomenex, United Kingdom) with a linear gradient from 75% Buffer A mixed with 25% Buffer B to 100% Buffer B over 36 min at a flow rate of 1 ml/min, and retention time of each peptide was recorded.

Additionally, circular dichroism (CD) analyses were carried out utilizing a JASCO J815 Spectropolarimeter (JASCO Inc., United States). Samples were measured within the range of 190–250 nm at 20°C. The parameters were set as: 200 nm/min scanning speed, a bandwidth of 1 nm, and 0.5 nm data pitch. Both peptide samples were dissolved in 10 nM ammonium acetate buffer and 50% TFE in 10 mM ammonium acetate buffer at a concentration of 100 μM. The percentage of the α-helix structure was predicted by the website K2D3^2^ (Louis-Jeune et al., 2012).

### *In vitro* Antimicrobial Assay

The antimicrobial activity of both natural and designed peptides was evaluated by MIC and MBC assays utilizing the broth dilution method. Microorganisms including *S. aureus* (NCTC10788), MRSA (NCTC 12493 and ATCC43300), *Escherichia coli* (*E. coli*) (NCTC 10418), *Pseudomonas aeruginosa* (*P. aeruginosa*) (ATCC 27853), *Candida albicans* (*C. albicans*) (NCYC 1467) and four clinical MRSA strains (DTMR8, DTMR24, DTMR37, and DTMR121) obtained at the First People’s Hospital of Zhenjiang, China were used. Molecular identification of these four clinical isolates was performed by PCR targeting *mecA* gene prior to use. After incubation of peptides (ranging from 1 μM to 512 μM, twofold serial dilution) and diluted microorganism cultures for 16 h, the absorbance values of the wells of the 96-well plates were detected at 550 nm with a Synergy HT plate reader (Biotech, United States) and the MICs were defined as the lowest concentration of peptides at which no apparent growth was detected. Ten microliters of culture in the wells were loaded onto Mueller-Hinton agar (MHA) medium and incubated at 37°C for another 18 h. The MBC of the peptide against a microbe was defined as the lowest concentration at which no evidence of colony growth was observed. Five replications were employed for all peptides with different concentrations and controls, and all measurements were made in triplicate.

### Antibiofilm Assay

Minimum biofilm eradication concentration (MBEC) assay was carried out with a modified microtiter plate method as described previously (Gao et al., 2017).

### Hemolysis Assay

Hemolytic property of peptides was measured using erythrocytes prepared from defibrinated horse blood (TCS Biosciences, Botolph Claydon, Buckingham, United Kingdom) as indicated previously (Wu et al., 2016).

### Cytotoxic Assay

Human keratinocyte cell line HaCaT and human bronchial epithelial cell line 16HBE were obtained from the ATCC. Cells were harvested and diluted to 5 × 10^4^ cells/ml using fresh medium. Ninety-nine microliters of diluted cells and 1 μl of peptides (final concentration ranging from 1 to 512 μM, twofold serial dilution, five duplicates) were mixed and seeded into 96-well culture plates. After incubation for 24 h, 10 μl CCK-8 was added into each well, followed by incubation at 37°C for 2 h. Absorbance of each well was determined, and the viabilities of cells were calculated as percentages of the control.

### *In vivo* Antimicrobial Assay

BALB/c mice (male, 6–8 weeks old) were purchased from Qinglongshan Laboratory Animal Company (Nanjing, China), and maintained under specific-pathogen-free conditions at 18–25°C and 50–60% humidity. All procedures involving animals were approved by the Animal Care and Use Committee of Nanjing University of Chinese Medicine and performed strictly according to the Guide for the Care and Use of Laboratory Animals. Peptides at 10.0 mg/kg and vancomycin at 20 mg/kg were intraperitoneally injected (i.p.) after mice being nasally inoculated with MRSA (1 × 10^7^ CFU in 20 μl PBS) for 1 h, subsequently. Twenty-four hours after administration, mice were euthanized. Mice trachea was cannulated, and 0.5 ml of cold PBS was infused intratracheally and withdrawn after left lung was ligated. This procedure was repeated three times, and consequently, a total volume of 1.2 ml of bronchoalveolar Lavage Fluid (BALF) was collected. Twenty micrograms of the left lung were homogenized in 0.5 ml of PBS for determining bacterial burden in the infected lungs. In addition, to measure the degree of pulmonary edema, the upper lobe of the left lung was excised and weighed immediately (wet weight). After being dried at 60°C for 72 h, weight of the lobe was measured (dry weight), and lung wet/dry ratio was calculated. The remaining tissue was fixed and stained with hematoxylin and eosin for pathologic observation, and the pathologic score for each mouse at a scale of 0–5 was calculated.

### *In vivo* Toxicities of Peptides

Mice were administered with 20.0 and 40 mg/kg of nigrocin-HL and nigrocin-HLM (i.p.) and a 7-day mortality was recorded.

### Statistical Analyses

Data analyzed are shown as mean ± standard deviations (SD). Multiple groups were compared with one-way analysis of variance and two groups with Dunnett’s test, using GraphPad Prism 7 (GraphPad Software, San Diego, CA, United States). For MBEC assay, viabilities of nigrocin-HL and nigrocin-HLM at each certain concentration were compared with two-tailed *t*-test. Statistical significance was set at *P* < 0.05.

### Accession Number

GenBank accession number of nigrocin-HL is MH167408^3^.

## Results

### “Shotgun” Cloning of Novel Nigrocin-HL Precursor-Encoding cDNAs From Skin Secretion-Derived cDNA Libraries of *H. latouchii*

**FIGURE 1:**
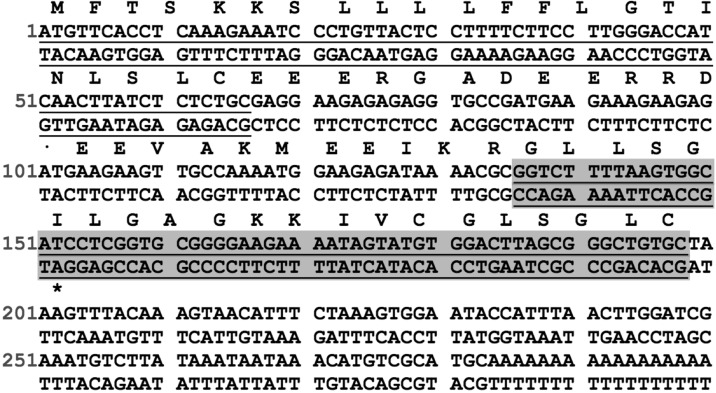
Nucleotide sequence of the cDNA cloned from skin secretion of *H. latouchii* and its predicted peptide sequence. The putative signal peptide (underlined), mature peptide (underlined and shaded) and stop codon (asterisk) are indicated.

A novel nigrocin peptide precursor-encoding cDNAs was cloned from the skin secretion- derived cDNA library of *H. latouchii*. The nucleic acid and translated amino acid sequence of the novel nigrocin-encoding precursor contained an open-reading frame (ORF) of 66 amino acid residues (Figure 1). The translated ORF consisted of a putative signal peptide (MFTSKKSLLLLFFLGTINLSLC) of 22 amino acid residues, a mature peptide of 21 amino acid residues, and an acidic spacer peptide between these two domains. This peptide precursor showed high similarity to a previously described peptide from the nigrocin family, named nigrocin-OG20 (Li et al., 2007).

### Depletion of “Rana box” Barely Reduced Antimicrobial Activity of Nigrocin-HL

To assess if the “Rana box” sequence was a key motif to maintain the bioactivity of the natural nigrocin-HL, we synthesized a shorter peptide (GLLGGILGAGKKIV) without the heptapeptide sequence. As suggested by MICs and MBCs listed in Table 1, the shorter analog showed similar activity against several strains of bacteria compared with the original peptide. In addition, both peptides displayed negligible potency against drug-resistant microbes, including *P. aeruginosa* (ATCC27853) and six different strains of MRSA. These data indicated that the “Rana box” motif was not indispensable for nigrocin-HL possessing antimicrobial activity.

**Table 1:** MICs and MBCs (mg/l) of nigrocin-HL and its analogs nigrocin-HLD and nigrocin-HLM against employed microorganisms.

Bacterial strains	Nigrocin-HL	Nigrocin-HLD	Nigrocin-HLM
*S. aureus* (NCTC10788)	62.70/250.81	42.43/169.73	2.94/5.89
*E. coli* (NCTC10418)	125.40/ND	84.87/ND	11.77/23.55
*C. albicans* (NCYC1467)	62.70/501.61	42.43/339.46	2.94/5.89
*P. aeruginosa* (ATCC27853)	ND/ND	ND/ND	47.08/47.08
MRSA (NCTC12493)	ND/ND	ND/ND	5.89/5.89
MRSA (ATCC43300)	ND/ND	ND/ND	1.47/5.89
DTMR8	ND/ND	ND/ND	1.47/2.94
DTMR24	501.61/ND	ND/ND	1.47/1.47
DTMR37	ND/ND	ND/ND	1.47/2.94
DTMR121	ND/ND	ND/ND	1.47/1.47

*ND, no detectable activity; all data were quoted in two decimal places.*

### Motif-Targeted Peptide Design

**FIGURE 2:**
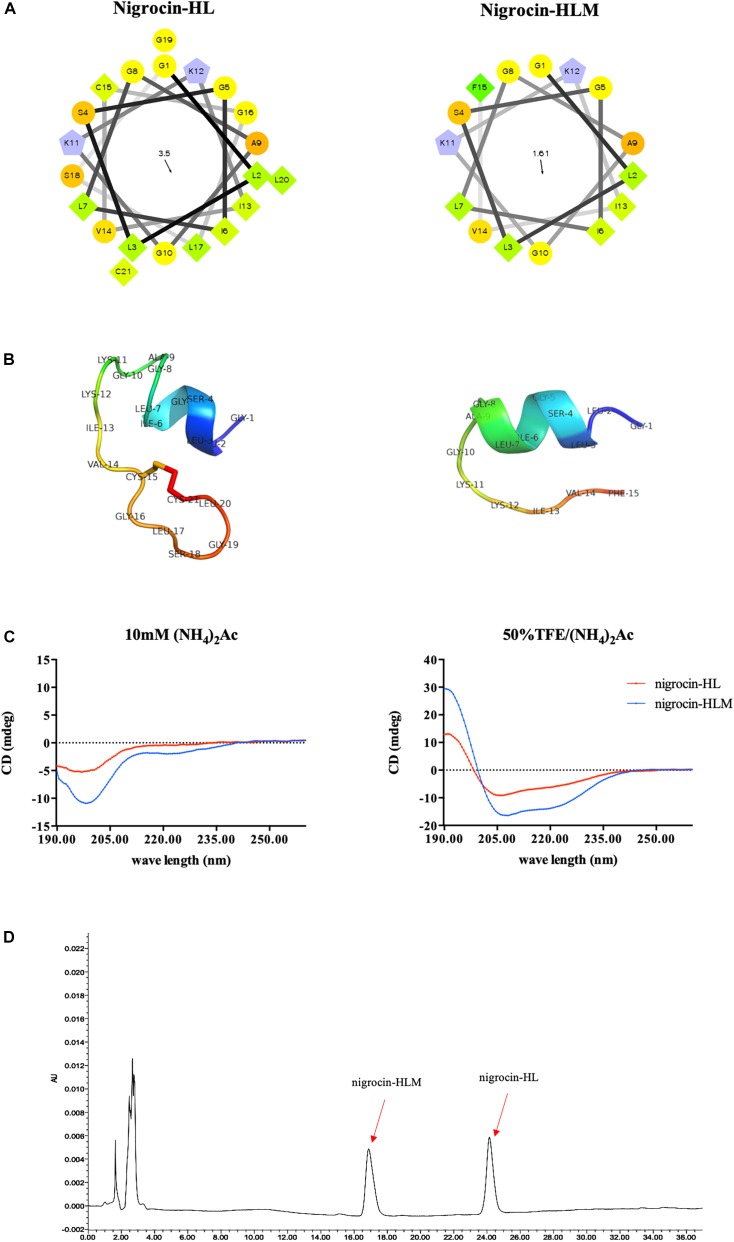
**(A)** Helical wheel plots of peptides were obtained from helical wheel projections, and the direction of summed vectors of hydrophobicity were indicated as arrows. **(B)** 3D models of peptides were firstly predicted by I-TASSER. Ramachandran plot of nigrocin-HL generated by PROCHECK showed that 76.9% of amino acid residues lied in the most favored regions and 23.1% in additional allowed regions. Evaluation of nigrocin-HLM by Ramachandran plot showed that 70% of residues in the most favored regions and 30% in additional allowed regions. Furthermore, the overall qualities of these 3D models were validated evident based on the results generated by PROSA. Specifically, the Z scores of nigrocin-HL and nigrocin-HLM were –3.45 and –1.22, respectively. **(C)** CD spectra of peptides (50 μM) were recorded in ammonium acetate buffer and in 50% TFE ammonium acetate buffer. **(D)** Hydrophobicity of nigrocin-HL and nigrocin-HLM were determined using RP-HPLC.

We modified the natural peptide by substituting the heptapeptide motif with an amidated phenylalanine residue, and an shorter analog named nigrocin-HLM was obtained (Figure 2A and Table 2). Our targeted modification, based on the predicted data, increased the helicity but decreased both the hydrophobicity and hydrophobic moment of the modified peptide. As shown in Figure 2B, three dimensional models of these peptides were predicted by I-TASSER, PyMOL and Swiss-PDB-Viewer and validated by PROCHECK and PROSA.

**Table 2:** The predicted physiochemical parameters and percentage of helical structure of nigrocin-HL, nigrocin-HLD, and nigrocin-HLM.

Peptides	Sequence	Helicity (%)	Hydrophobicity (H)	Hydrophobic moment (μH)	Net charge (z)
Nigrocin-HL	GLLSGILGAGKKIVCGLSGLC	31.28	0.698	0.465	+2
Nigrocin-HLD	GLLSGILGAGKKIV	52.32	0.586	0.533	+2
Nigrocin-HLM	GLLSGILGAGKKIVF-NH_2_	50.67	0.667	0.388	+3

In addition, the hydrophobicity of nigrocin-HL and nigrocin-HLM were determined using RP-HPLC (Figure 2D), and the retention times suggested that nigrocin-HLM displayed reduced hydrophobicity compared with the natural peptide. Besides, the secondary structure of peptides was predicted by CD spectrum (Figure 2C). In contrast with the natural peptide, the intensity of band at about 208 nm and 225 nm for nigrocin-HLM in 50% TFE dramatically augmented, which coincided with the predicted data. As shown in Table 2, the helicity of nigrocin-HL was 31.28%, and it significantly increased to 50.67% for nigrocin-HLM after modification.

### *In vitro* and *in vivo* Antimicrobial Activities of Nigrocin-HL and Its Modified Analog Nigrocin-HLM

The MICs and MBCs of nigrocin-HL and nigrocin-HLM against microorganisms tested are summarized in Table 1. Nigrocin-HLM was potent against the growth of *S. aureus*, *E. coli* and *C. albicans* with inhibition being increased over 10-fold, comparing to the natural peptide. Consistent with MICs, nigrocin-HLM displayed dramatically enhanced bactericidal activities against these three representative bacteria as demonstrated by precipitously declined MBCs. The modified peptide possessed relatively strong bioactivity against antibiotic *P. aeruginosa* (ATCC27853) with MIC and MBC at 32 μM (47.08 mg/l). Even more intriguing, nigrocin-HLM showed potent activity against MRSA (NCTC12493, ATCC43300, four clinical isolates DTMR8, DTMR24, DTMR37, and DTMR121) with MIC of 4 μM (5.89 mg/l) against NCTC12493 and 1 μM (1.47 mg/l) against the rest. In sharp contrast with the natural peptide, nigrocin-HLM also showed bactericidal effect against all the tested MRSA with MBC not higher than 6 mg/l.

**FIGURE 3:**
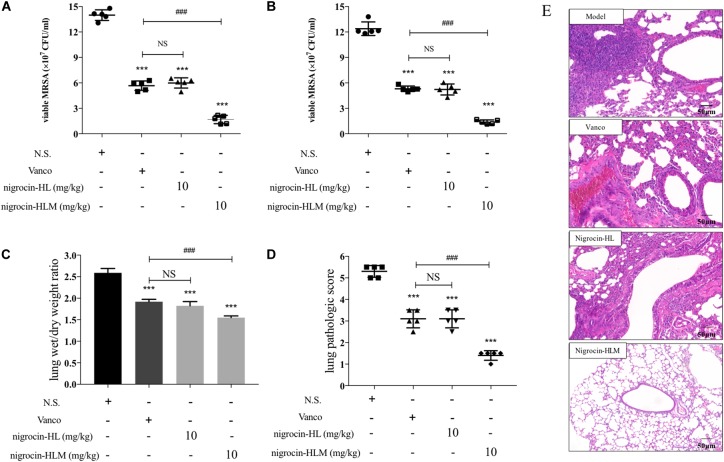
Efficacy of peptides in the MRSA-infected pneumonia mice model. Mice were nasally inoculated with 1 × 10^7^ CFU MRSA. One hour after infection, normal saline, 20 mg/kg vancomycin and both peptides at 10 mg/kg were administered by i.p. injection. The number of CFU in the lung **(A)** or in the BALF **(B)** was calculated from the number of colonies growing on MHA plates. **(C)** Effects of nigrocin-HL and nigrocin-HLM on lung inflammation caused by MRSA were analyzed by lung wet/dry ratio. Left lung of each infected mice was stained with HE and analyzed microscopically. **(D)** The pathologic score of lungs at a scale of 0–5 was calculated for each mouse. Data was shown as mean ± SD. Horizontal bars refer to standard deviations. ^∗∗∗^*P* < 0.001 versus the model group (normal saline, NS), and ^###^*P* < 0.001 versus the vancomycin (vanco) group. NS, not statistically significant. **(E)** Lung histopathologic changes of MRSA-induced pneumonia in mice. The magnification is ×200. The bar length represents 50 μm.

As shown in Figures 3A,B, nigrocin-HLM at 10 mg/kg significantly ameliorated the lung infection caused by MRSA, as evidenced by reduced viable bacteria in lung and BALF of infected mice. Besides, we evaluated the pathological changes by HE staining (Figures 3D,E) and determining the wet/dry ratio of lungs (Figure 3C). Results showed that nigrocin-HLM at 10 mg/kg markedly ameliorated pulmonary inflammation and edema caused by MRSA, which was even better than vancomycin at 20 mg/kg. Meanwhile, nigrocin-HL showed similar trend but relatively weaker potency.

### Biofilm Eradication Activity of Nigrocin-HL and Its Analog Against MRSA Biofilm

**FIGURE 4:**
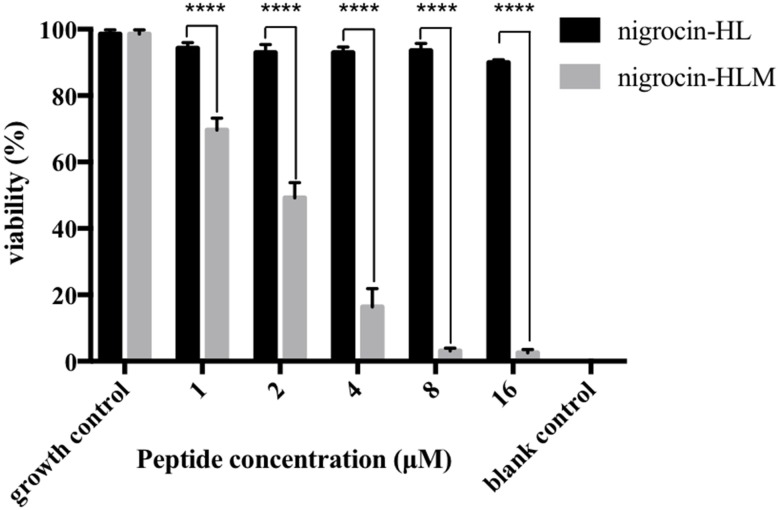
Minimum biofilm eradication concentration (MBEC) of peptides against mature MRSA biofilm. All data represent means ± SD from three independent experiments performed in triplicate (^∗∗∗∗^*P* < 0.0001 versus the viability of the corresponding concentration of nigrocin-HL).

As shown in Figure 4, nigrocin-HLM eradicated MRSA biofilm with a MBEC concentration of 8 μM (11.77 mg/l), in sharp contrast with nigrocin-HL which displayed negligible inhibition of biofilm formation up to 16 μM (31.35 mg/l).

### *In vitro* and *in vivo* Toxicities of Nigrocin-HL and Nigrocin-HLM

**FIGURE 5:**
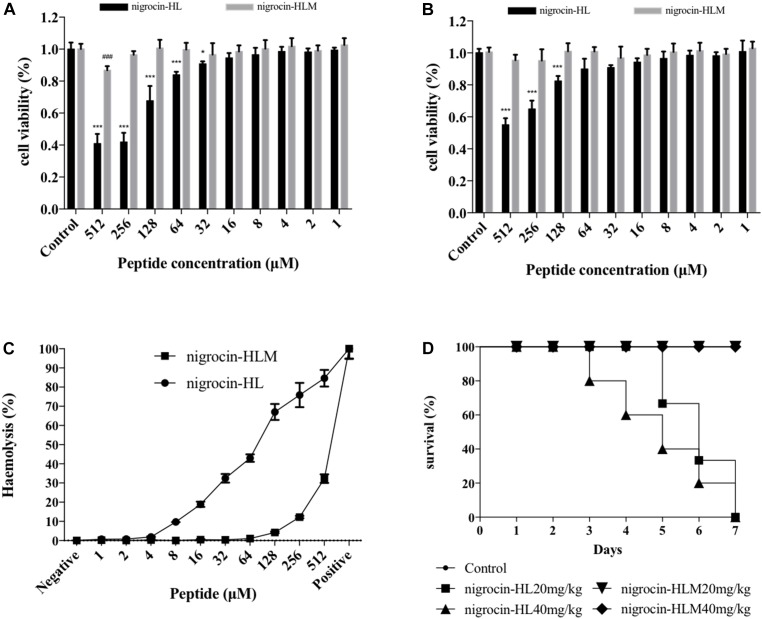
Toxicities of nigrocin-HL and its analog nigrocin-HLM *in vitro* and *in vivo*. Cytotoxicity of nigrocin-HL and nigrocin-HLM against **(A)** HaCaT and **(B)** 16HBE. Living cells after treatment with peptides were measured utilizing CCK8 assays, and viability was calculated relative to the untreated control. **(C)** Hemolysis of nigrocin-HL and nigrocin-HLM was determined against horse erythrocytes. All data represent mean ± SD from three independent experiments performed in triplicate (^∗^*P* < 0.05, ^∗∗∗^*P* < 0.001, compared with nigrocin-HL control group; ^###^*P* < 0.001, compared with nigrocin-HLM control group). **(D)** *In vivo* toxicities of these two AMPs. Survival rate of mice in 7 days after single i.p. injection of nigrocin-HL and nigrocin-HLM (20 and 40 mg/kg) was recorded.

Despite of potent activities of these two AMPs, toxicities of them remain to be assessed. Cytotoxicity of nigrocin-HL and nigrocin-HLM were determined using human keratinocyte cell line HaCaT and human bronchial epithelial cell line 16 HBE. Cell growth inhibition of HaCaT and 16HBE occurred with nigrocin-HL dosages as low as 32 μM, which was not observed in nigrocin-HLM up to 512 μM (Figures 5A,B). In addition, hemolytic capacities of these two AMPs were measured against horse erythrocytes. As shown in Figure 5C, whereas the natural peptide induced obvious hemolysis at its MICs against tested microorganisms, the modified nigrocin-HLM showed negligible hemolytic activity even at the concentrations for it to exert bactericidal effects. In fact, nigrocin-HLM was found to be devoid of hemolytic activity up to 128 μM.

Furthermore, acute toxicities of nigrocin-HL and nigrocin-HLM *in vivo* were assessed in BALB/c mice via i.p. injection of different doses of peptides. Survival rates were recorded in 7 days. After single injection of nigrocin-HL at 20 and 40 mg/kg, mice all died from day 3 to day 7. In contrast, no mortality was observed in the two nigrocin-HLM groups within 7 days (Figure 5D).

## Discussion

As a hospital-acquired antibiotic-resistant microorganism, MRSA has causes substantial morbidity and mortality worldwide (DeLeo et al., 2010). The antibiotic resistance crisis caused by some developed bacteria, represented by MRSA, has outpaced the attempts to develop new antibiotics. Under this circumstance, AMPs have been considered as promising alternative candidates against infection especially in the post-antibiotic era (Fjell et al., 2011). In this present study, we isolated and identified an AMP named nigrocin-HL from skin secretion of the broad-folded frog, *H. latouchii*. This novel AMP bears a heptapeptide motif (CGLSGLC) named “Rana box,” which is shared throughout anuran AMPs including brevinins, gaegurins, esculentins, and nigrocins (Ponti et al., 1999; Kozic et al., 2015). “Rana box” is mostly constituted by two flanking cysteine residues separated by other four or five residues, with the second cysteine being at the C-terminus, it forms a cyclic disulfide bridge (Iwakoshi-Ukena et al., 2011a). This similarity of AMPs derived from different species indicates that they are likely to have an evolutionary origin, the significance of this characteristic structure on potency of AMPs against microbes remains to be elucidated, though. Previous paper reported that deletion of “Rana box” radically abolished the antimicrobial activity of AMP, while others dismissed its function, as the elimination or replacement of cysteine residue did not affect potency of AMPs (Kwon et al., 1998; Vignal et al., 1998; Abraham et al., 2015).

To confirm whether the “Rana box” sequence is indispensable for nigrocin-HL to exert its action, we synthesized a shorter peptide nigrocin-HLD without the motif. *In vitro* activity assays suggested that the shorter analog displayed similar efficiency to the natural peptide against several microbes including Gram- positive *S. aureus*, yeast *E. coli* and Gram- negative *C. albicans*. In addition, these two peptides both displayed negligible activity against drug-resistant *P. aeruginosa* and several strains of MRSA except for nigrocin-HL at 256 μM (501.61 mg/l) inhibiting the growth of a clinical isolated MRSA DTMR24. These data indicated that the peptide possessed moderate antimicrobial activity regardless of whether the highly conserved motif “Rana box” existed or not.

Thus, we modified the natural nigrocin-HL into a shorter peptide nigrocin-HLM by substituting the motif with a hydrophobic aromatic phenylalanine residue and amidating the C-terminus of it. As a result, nigrocin-HLM displayed much stronger activities against broad spectrum of microorganisms than nigrocin-HL as evidenced by striking decrease of both MIC and MBC. Most strikingly, “Rana box” substitution with phenylalanine residue enabled nigrocin-HLM to exert potent bacteriostatic as well as bactericidal activity against seven MRSA strains including five clinical isolates. We further evaluated antimicrobial activity of peptides in an MRSA-induced pneumonia mice model, and results indicated that nigrocin-HLM showed excellent potency against MRSA *in vivo*, which was even more efficient than vancomycin at 20 mg/kg. Besides, since toxicity is one of the major obstacles for AMPs being applied clinically, both toxicity of peptides *in vitro* and acute toxicity *in vivo* were assessed. Consistent with our expectation of modification, nigrocin-HLM displayed much less toxicity than the natural peptide.

To the best of our knowledge, a peptide with potent antimicrobial efficacy and high selectivity spontaneously should possess optimum balance between various parameters of it, including hydrophobicity, secondary structure and cationicity, etc. (Wimley, 2010). The biological activity of AMPs is strongly correlated with cationicity of them owing to the fact that AMPs are initially attracted to the surface of bacterial cell membranes by the electrostatic force between positively charged residues within AMPs and anionic phospholipids and lipopolysaccharides of the bacterial cell membrane. In consequence, AMPs with more cationic residues should possess stronger antimicrobial activity theoretically. However, adding cationic residues above the optimum charge of magainin 2 remarkably decreased its selectivity for microbial cells and resulted in increased hemolysis (Dathe et al., 2001). Substitution with a phenylalanine residue preserved the cationicity of nigrocin-HLM, which helped to maintain its activity but no more toxicity simultaneously. In addition, secondary structure and hydrophobicity of peptides have been considered as other key properties to cooperatively govern the antimicrobial potency and hemolytic capacity of them. Earlier, it was reported that moderate hydrophobicity and more stable helical structure of the 14-helical β-peptide elicited higher antifungal activity and higher selectivity toward *C. albicans* (Lee et al., 2014). It has been widely accepted that a proper balance between α-helicity and hydrophobicity enhances the potency of AMPs to penetrate through bacterial membranes, resulting in membrane disruption and cell death in consequence (Hwang et al., 2013). In the study described herein, nigrocin-HLM displayed higher helicity and lower hydrophobicity comparing with the natural peptide, as demonstrated by CD spectra analysis and predicted data, respectively. Although the helical-forming structure “Rana box” was eliminated, the helicity of the modified peptide significantly increased because of shorter sequence in general. Besides, two hydrophobic leucine residues were removed systematically when the “Rana box” sequence was substituted with one hydrophobic phenylalanine residue, which markedly reduced the hydrophobicity of nigrocin-HLM as evidenced by the predicted data and further RP-HPLC confirmation. Previous paper claimed that higher or lower hydrophobicity beyond an optimum hydrophobicity window of the 26-residue peptide V13K_L_ remarkably decreased its antimicrobial activity (Chen et al., 2007). Nigrocin-HLM, consistent with this theory, possessed decreased hydrophobicity but showed significantly improved and broadened antimicrobial activity in contrast with the natural AMP, which indicated that our modification helped to modify the hydrophobicity to an optimum window. And in accordance with the previous paper, we speculated that weaker potency of the natural nigrocin-HL might be attributed to stronger peptide dimerization in solution caused by high hydrophobicity of it, which prevented access to the membranes of bacteria (Chen et al., 2007). Meanwhile, the natural peptide showed much stronger toxicity either *in vitro* or *in vivo*, and there was a substantial chance that its toxicity correlated with high hydrophobicity-induced self-association because strong self-association of AMPs enables them to penetrate through bacterial membranes but interfere eukaryotic cell membranes as well (Huang et al., 2010). Previously, C-terminal amidation of PMAP-23 was reported to possess more rapid bactericidal kinetics and more helical structure of it in the bacterial membrane environment than the same concentration of PMAP-23C because of the increased cationic charge (Kim et al., 2011). In addition, the amidation of C-terminal residue might be responsible for the stabilization of the secondary helical structure of mastoparans that is richer than its congener (da Silva et al., 2014). Meanwhile, Irazazabal et al. (2016) suggested that the increase of net charge by +1 of [I^5^, R^8^] MP-amide might compensate and overcome the lower level of coil-to-α-helix transition compared with the non-amidated peptide. Taken these into consideration, C-terminal amidation of the modified peptide may also facilitate to its antimicrobial activity by stabilizing membrane interactive peptide structure and improving the partial charge of the modified analog. Last but not least, a phenylalanine residue might also provide extra membrane- binding driving force for nigrocin-HLM since it contains an aromatic ring (Lee et al., 2013; Datta et al., 2016).

Prevalently, a cluster of microbes could form biofilms by attaching to surfaces and producing extracellular polysaccharides, which enables bacteria grow in a physiologically distinct way and causes severe infections. MRSA-caused infection is one of the most worrisome problem for public health not only because of its multidrug resistance but also owing to its strong tendentiousness in forming biofilm. Furthermore, MRSA growing in a biofilm are more recalcitrant to antibiotics than growing in a planktonic state because of their low metabolic activity, which enables them resistant to most antibiotics targeting metabolically active cells (Kanthawong et al., 2012). The modified peptide nigrocin-HLM at 8 μM eradicated MRSA-biofilm, in sharp contrast with the natural nigrocin-HL up to 16 μM showing negligible inhibition of biofilm formation. We speculate that nigrocin-HLM inhibits formation of MRSA-biofilm by permeabilizing and/or forming pores within the cytoplasmic membrane. Our modification of the natural nigrocin-HL empowered it with the activity of eradicating MRSA-biofilm, whereas there have not been any papers reporting antibiofilm potency of nigrocins previously.

To summarize, we firstly dismissed the indispensable role of the “Rana box” sequence in maintaining the potency of nigrocin-HL. Subsequently, we optimized the representative parameters of nigrocin-HLM including hydrophobicity, helicity and cationicity by substituting the “Rana box” sequence within the natural peptide with an amidated phenylalanine, which facilitated its activity against several strains of microbes and notably, our modification even broadened its potency against antibiotic-resistant microorganisms including MRSA and *P. aeruginosa*. Besides, the analog exhibited high potency to inhibit MRSA not only in planktonic but biofilm state as well. Most importantly, the modification significantly attenuated toxicity of nigrocin-HLM both *in vitro* and *in vivo*, raising the possibility of it being applied clinically. With the encouraging antibacterial activity and negligible toxicity, nigrocin-HLM should be considered as a new promising therapeutic candidate against a broad range of microbes, which may contribute to counter the antibiotic-resistance threat. Meanwhile, other aspects like administration route is another prospective where nigrocin-HLM is yet to be exploited to its utmost.

## Author Contributions

KB, CM, LW, XX, MH, MZ, and TC contributed to experimental design. KB, XY, and WY performed the experiments and collected data. The analysis of data was conducted by KB, LW, and XX. The manuscript was written, revised and edited by KB, MH, and XX.

## Conflict of Interest Statement

The authors declare that the research was conducted in the absence of any commercial or financial relationships that could be construed as a potential conflict of interest.

## Footnotes

1 http://heliquest.ipmc.cnrs.fr

2 http://cbdm-01.zdv.uni-mainz.de/~andrade/k2d3//

3 https://www.ncbi.nlm.nih.gov/nuccore/MH167408
