# Supplementary figures and images for: Modification Targeting the “Rana Box” Motif of a Novel Nigrocin Peptide From Hylarana latouchii Enhances and Broadens Its Potency Against Multiple Bacteria
Source: Front Microbiol. 2018 Nov 28;9:2846. doi: 10.3389/fmicb.2018.02846 (PMC6280737; doi:10.3389/fmicb.2018.02846)

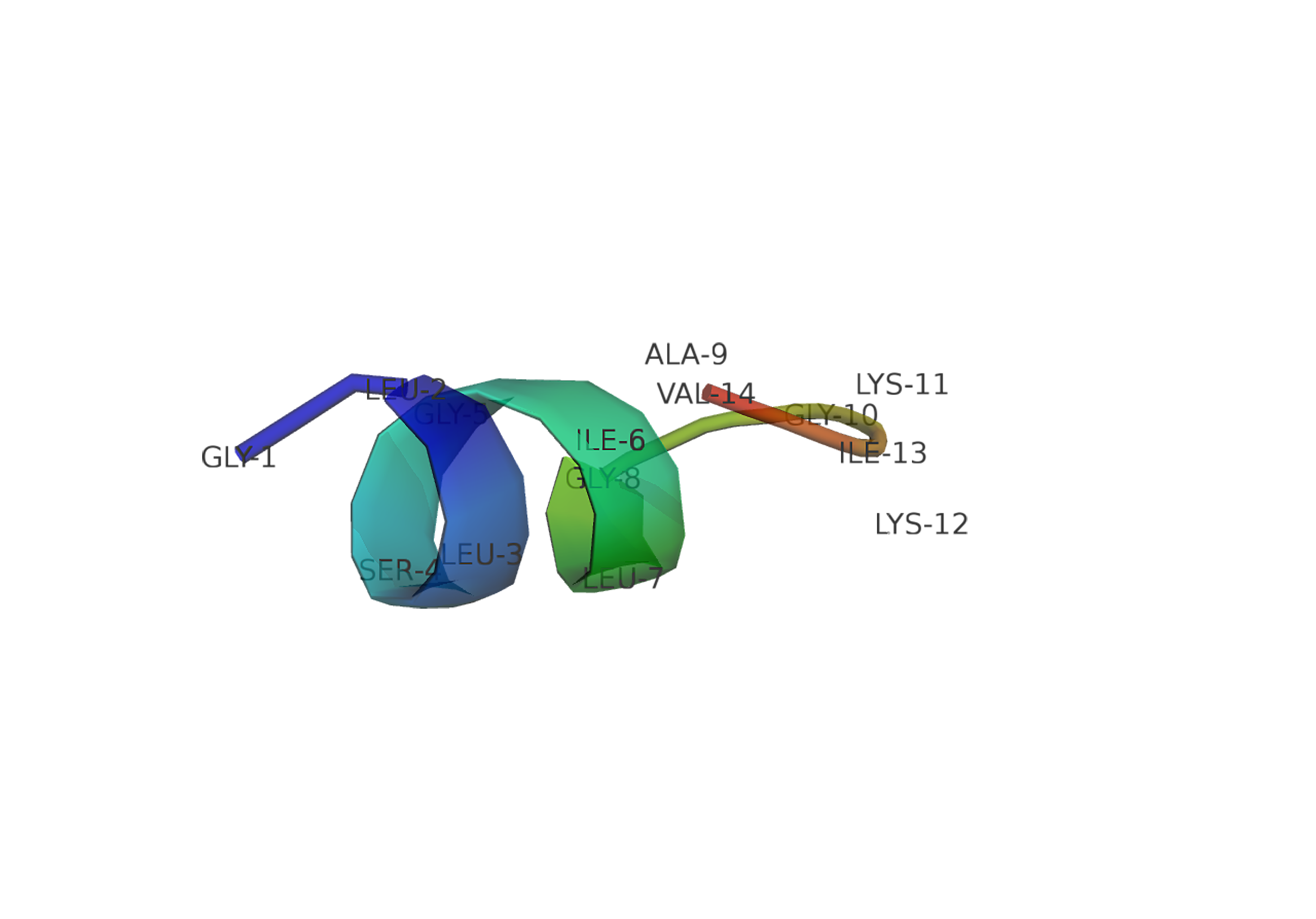

Supplement: Supplementary file 1 [file Image_1.TIF]
